# Honors Conferred upon American Physicians

**Published:** 1858-01

**Authors:** 


*Honors conferred upon American Physicians.*—In the Med. & Surg. Reporter, we have a list of the honors conferred by the Emperor of Russia upon American physicians who served in the late war between Russia and the Allied Powers.

"Dr. E. B. Turnipseed, now of New York, a native of South Carolina, has recently received from the Emperor, through Baron Stœckel, Russian Minister at Washington, the decoration of the Russian order of St. Anne of the third class, accompanied by two medals attached to the ribbons of the orders of St. George and St. Andrew, in acknowledgment of his services during the war. The Emperor has also conferred the orders of St. George and St. Andrew upon Drs. Harris, Holt, Smith, Eldridge, Johnson, and Matthews, all American physicians, who were associated with Dr. Turnipseed.

"The cross of the order of St. Anne is a neat piece of workmanship, set in gold an enamel. The decoration of St. George is the highest military insignia in the empire, permitted only to those whose lives have been risked in the service of the Emperor. The St. Andrew ribbon is the highest civil honor. The above facts we gather from the *N. Y. Daily Times.*"

We have certainly every reason to be proud of the appreciation which the services of our countrymen met with, during the last war, from Russia and the French. The restless spirit of a true American is so powerful that it cannot be subdued even by the studies and experiences of medical life, and the opportunities open to the medical man for gratifying this propensity, are so few, that a war such as the last, is a perfect Godsend. We see this exemplified in the numberless applications for appointments in the army and navy, where, on this account, the examinations have been made so rigid, and the standard of merit has been placed so high, that a comparatively small proportion are accepted. Wherever our countrymen go, however, they leave the impress of characteristic energy and zeal in doing what they have to do, as is exemplified by the gratifying testimonials which have been presented to them by the Russian Emperor. F.

---

*Novel Method of Extracting a Foreign Body from the Œsophagus.*—We have before us, in the pages of the Boston Medical Journal, an account of an extremely ingenious and novel method of extracting a foreign body from the œsophagus, by Dr. David Rice. "Mrs. Field, a lady aged 70, while eating chicken soup, accidentally swallowed a piece of bone the size of an American quarter of a dollar, cut into a triangular form. The bone longed in the œsophagus, about two inches below the top of the sternum. Thinking that it might fall into the stomach, she neglected to apply for surgical aid until the fifth day after the accident. In the mean time, she had swallowed neither food nor drink, both regurgitating back into the mouth with every attempt to do so."

The Dr. was called on the fifth day, but was unable to remove the foreign substance by any instrument which he had at his command. He finally took a piece of sponge of such a shape, as when dry, to fill about half of the tube, and introduced it rapidly in a dry state, then, by introducing a little water into the mouth, the sponge became moistened, and enlarged it to twice its natural size, completely filling the gullet. On drawing it out the bone was brought with it, much to the gratification of patient and practitioner.

There is a certain readiness of invention and expedient which is necessary to a surgeon, and without which he will often be nonplused and harrassed. The same means cannot be applied to every case, and common sense, with a share of ingenuity, frequently is all that is necessary to overcome difficulties which seem to be very great. It is desirable to have in our minds the expedients which have been resorted to by others in difficult cases, for we are liable at any time, to have a case to which they are precisely applicable. We conceive that this mode of swabbing the gullet from below upward, by introducing a dry sponge below a foreign body, allowing it to imbibe moisture and then withdrawing it, might be applicable to a great many cases where the substance could not be removed by other means. F.

